# Supplementary material for: New Insights into Human Cytomegalovirus pUL52 Structure
Source: Viruses. 2021 Aug 18;13(8):1638. doi: 10.3390/v13081638 (PMC8402748; doi:10.3390/v13081638)
Supplement: Supplementary file 1 [file viruses-13-01638-s001.zip › viruses-1303839-supplementary.pdf]

New insights into human cytomegalovirus pUL52 structure

Clotilde Muller[1], Sophie Alain[1,2], Claire Gourin[1], Thomas F. Baumert[3], Gaëtan Ligat[3, †,*], Sébastien Hantz[1,2, †,*]

[1]Univ. Limoges, INSERM, CHU Limoges, RESINFIT, U1092, F-87000 Limoges, France

[2]CHU Limoges, Laboratoire de Bactériologie-Virologie-Hygiène, National Reference Center for Herpesviruses (NRCHV), F-87000 Limoges, France

[3]Institut de Recherche sur les Maladies Virales et Hépatiques, Université de Strasbourg, 67000 Strasbourg, France

†These authors contributed equally

*Corresponding author:

gligat@unistra.fr (G.L.); sebastien.hantz@unilim.fr (S.H.)

SUPPLEMENTARY DATA

Table S1: Sequences used for alignment of pUL52 homologs.

Identification	Virus	Protein	Lenght residues	Accession number
PrV	Suid alphaherpesvirus 1	pUL32	470	>YP_068335.1
BoHV-1	Bovine alphaherpesvirus 1	pUL32	601	>NP_045326.1
HSV-1	Human herpesvirus 1	pUL32	596	>NP_044634.1
GaHV-1	Gallid alphaherpesvirus 1	pUL32	582	>YP_182361.1
GaHV-2	Gallid herpesvirus 2	pUL32	641	>NP_057791.1
HSV-2	Human herpesvirus 2	pUL32	598	>NP_044502.1
GaHV-3	Gallid alphaherpesvirus 3	pUL32	626	>NP_066864.1
VZV	Human alphaherpesvirus 3	pUL32	585	>NP_040149.1
CyCMV	Cynomolgus cytomegalovirus	Cy83	552	>YP_009337502.1
RCMV	Rat cytomegalovirus Maastricht	pR52	530	>NP_064158.1
RhCMV	Macacine betaherpesvirus 3	Rh83	551	>YP_068176.1
MCMV	Murid betaherpesvirus 1	M52	517	>AWV68147.1
HCMV	Human betaherpesvirus 5	pUL52	668	>DAA00157.1
HHV6-A	Human betaherpesvirus 6A	U36	484	>NP_042929.1
HHV6-B	Human betaherpesvirus 6B	Virion protein	484	>NP_050217.1
HHV7	Human betaherpesvirus 7	pUL32	485	>YP_073776.1
EBV	Human herpesvirus 4	pUL32	525	>NP_039846.1
SaHV-2	Saimiriine gammaherpesvirus 2	unnamed protein	436	>NP_040270.1

Table S2: Primers used for *UL52* amplification and sequencing.

Primers	Sequence 5′ → 3′
UL52-2 For	GCACGCGCACCCGCGTCA
UL52-3 Rev	GTGGTGTTGGTCGCATTGG
UL52-4 For	GCTTCGGCCGCCTGCGACCT
UL52-4_5 Rev	CGAAGCGGTCGCGGCTAGGTG
UL52-5 For	TGGGCCGGCACCGGCGTCAT
UL52-6 Rev	TGCGGGTCGCAGAAAAAGTG
UL52-7 For	CGTCAGGCAGGCGTCACGGG
UL52- Int1	CCAGAAACGGCACCCGCTGC
UL52- Int2	GGGTGCTCGCGGAAGATGTC
UL52- Ext1	TTACCCGGCGCCGACTCGTC
UL52- Ext2	GCCGTCCTCGGCGTGCATAA

Table S3: Primers used for HCMV-BAC recombinant viruses construction.

Mutation	Sens	5′ → 3′
UL52 C200S	For	ACTGCGGCGACTGTGCGCGCCGCTGGCCATCGACACGCGCTCCA ACCTATGCGCCATCATCAGTAGGGATAACAGGGTAATCGATT
	Rev	AGTCCTGTTTGAGGCAGATGCTGATGATGGCGCATAGGTTGGA GCGCGTGTCGATGGCCAGCGGCCAGTGTTACAACCAATTAACC
UL52 C203S	For	ACTGTGCGCGCCGCTGGCCATCGACACGCGCTGTAACCTATCCG CCATCATCAGCATCTGCCTTAGGGATAACAGGGTAATCGATT
	Rev	TCTGGTCGCAGTCCTGTTTGAGGCAGATGCTGATGATGGCGGAT AGGTTACAGCGCGTGTCGAGCCAGTGTTACAACCAATTAACC
UL52 C226S	For	GGACTGCGACCAGAGCTGGCTCCTCGAGTACAGCTTGCTGTCCT TCAAATGCAGTTACGCGCCTAGGGATAACAGGGTAATCGATT
	Rev	GCGTGCTGAGCGCCGCACGGGGCGCGTAACTGCATTTGAAGGA CAGCAAGCTGTACTCGAGGAGCCAGTGTTACAACCAATTAACC
UL52 C229S	For	CCAGAGCTGGCTCCTCGAGTACAGCTTGCTGTGCTTCAAATCCA GTTACGCGCCCCGTGCGGCTAGGGATAACAGGGTAATCGATT
	Rev	TGATGATGAGCGTGCTGAGCGCCGCACGGGGCGCGTAACTGGA TTTGAAGCACAGCAAGCTGTGCCAGTGTTACAACCAATTAACC
UL52 H276A	For	GTTCCGACACCACGTTCTCACGGTCTTCGATTTCCACCTGGCCTT TTTCATCAATCGTTGCTTTAGGGATAACAGGGTAATCGATT
	Rev	CGTCGCCCACTTGTTTTTCAAAGCAACGATTGATGAAAAAGGCC AGGTGGAAATCGAAGACCGGCCAGTGTTACAACCAATTAAC
UL52 C282S	For	CACGGTCTTCGATTTCCACCTGCACTTTTTCATCAATCGTTCCTTT GAAAAACAAGTGGGCGATAGGGATAACAGGGTAATCGA
	Rev	CATTCTCGTTATCAACCGCGTCGCCCACTTGTTTTTCAAAGGAAC GATTGATGAAAAAGTGCAGCCAGTGTTACAACCAATTAACC
UL52 C459S	For	CGTACCCGCTTTTTTTACCAAGAACCAAACCAGTACCGTGTCCCT GCTGTGCGAACTCATGGCTAGGGATAACAGGGTAATCGATT
	Rev	CGTTATCGTAATAGGAGCAGGCCATGAGTTCGCACAGCAGGGA CACGGTACTGGTTTGGTTCTGCCAGTGTTACAACCAATTAACC
UL52 C462S	For	TTTTTTTACCAAGAACCAAACCAGTACCGTGTGTCTGCTGTCCGA ACTCATGGCCTGCTCCTATAGGGATAACAGGGTAATCGATT
	Rev	GCAGGACGACGTTATCGTAATAGGAGCAGGCCATGAGTTCGGA CAGCAGACACACGGTACTGGGCCAGTGTTACAACCAATTAACC
UL52 H567A	For	CCACGTCCTGCGTCAGGCAGGCGTCACGGGCATCTACAAGGCCT TTTTCTGCGACCCGCAGTGTAGGGATAACAGGGTAATCGAT
	Rev	TGACGCGGATGTTGCCGGCGCACTGCGGGTCGCAGAAAAAGGC CTTGTAGATGCCCGTGACGCGCCAGTGTTACAACCAATTAACC
UL52 C570S	For	GCGTCAGGCAGGCGTCACGGGCATCTACAAGCACTTTTTCTCCG ACCCGCAGTGCGCCGGCAATAGGGATAACAGGGTAATCGATT
	Rev	CCTCGTTGGTGACGCGGATGTTGCCGGCGCACTGCGGGTCGGA GAAAAAGTGCTTGTAGATGCGCCAGTGTTACAACCAATTAACC

UL52 C574S	For	CGTCACGGGCATCTACAAGCACTTTTTCTGCGACCCGCAGTCCG CCGGCAACATCCGCGTCACTAGGGATAACAGGGTAATCGATT
	Rev	CGAAGAGCACGGCCTCGTTGGTGACGCGGATGTTGCCGGCGGA CTGCGGGTCGCAGAAAAAGTGCCAGTGTTACAACCAATTAACC

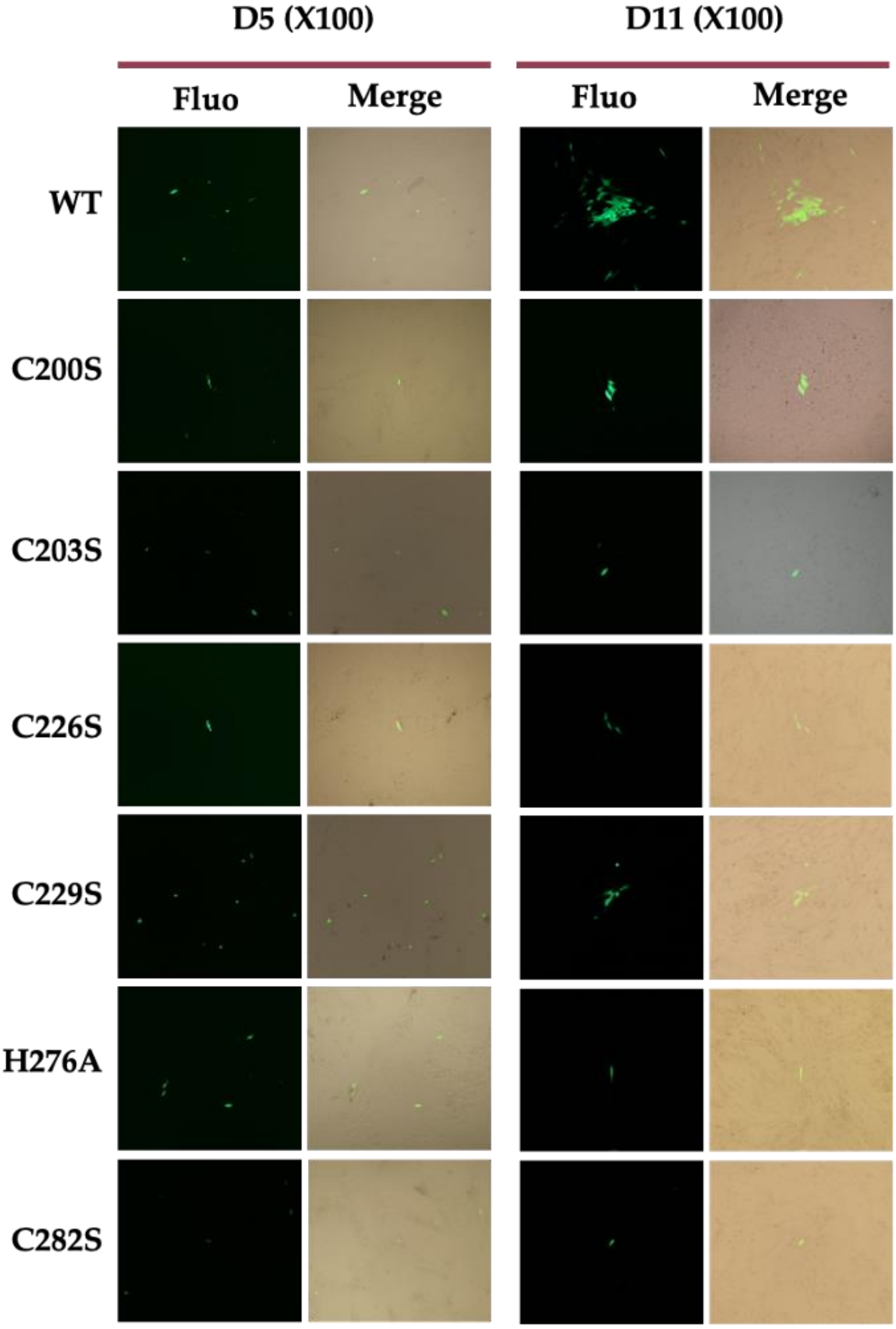

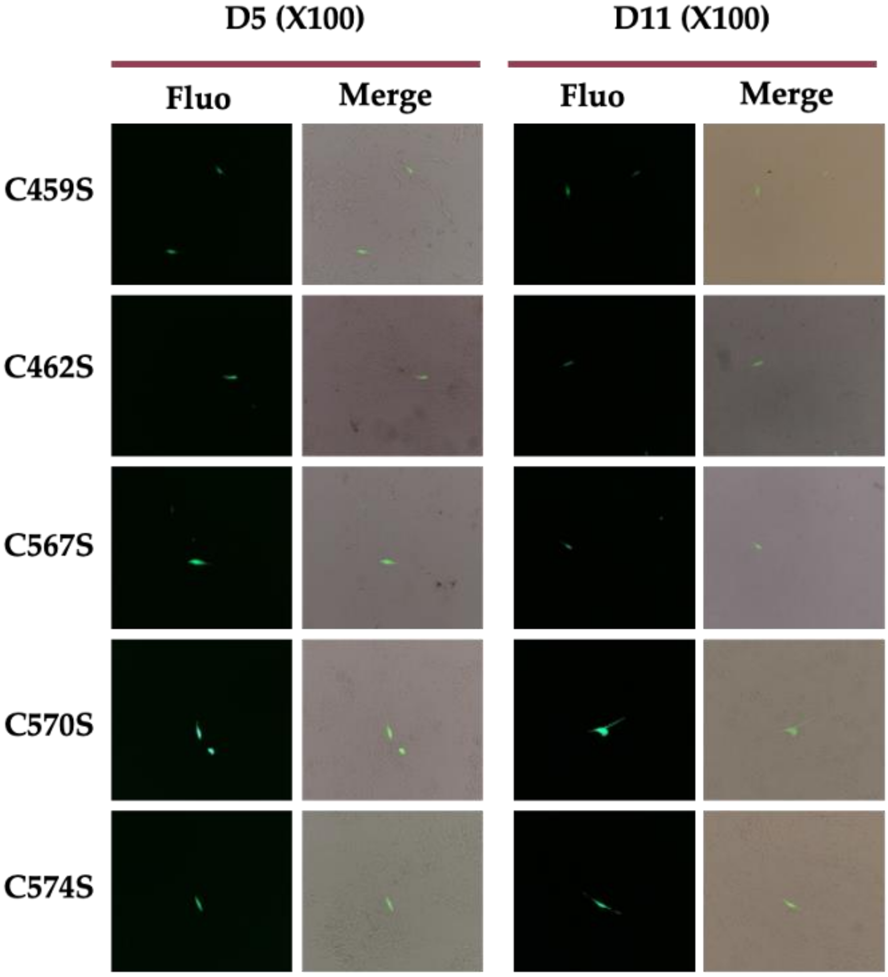

Figure S1. Plaque formation assay in MRC-5 cells after transfection of HCMV-BAC-*UL52* WT (AD169) or recombinant virus strains for the conserved region I, II and III. Green fluorescent foci were observed with the wild-type HCMV-BAC-*UL52* and single infected cells were observed with the other recombinant viruses. Eleven days after transfection of human fibroblasts, we observed no cytopathic effect for the different mutants except the recombinant strain AD169-UL52-C229S and AD169-UL52-C570S. These mutations dramatically impaired viral replication and propagation in cell-culture.

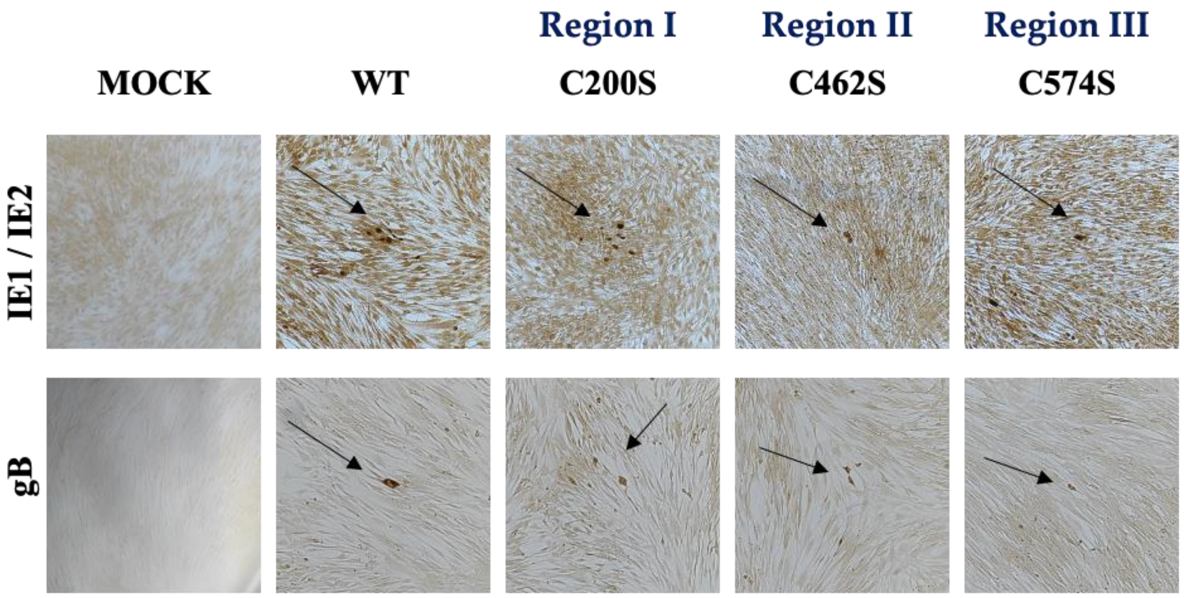

Figure S2. pUL52 is not required for viral late gene expression. MRC-5 were transfected with HCMV-BAC WT or one the mutant of each conserved region. Day 5 post-transfection, immunostaining was performed for early (IE1/IE2) and late (gB) viral proteins (black arrows). (Magnification: 100X).
